# Performance analyses of highly efficient inverted all-perovskite bilayer solar cell

**DOI:** 10.1038/s41598-023-35504-x

**Published:** 2023-05-22

**Authors:** Alireza Gholami-Milani, Sohrab Ahmadi-Kandjani, Babak Olyaeefar, Mir Hojjat Kermani

**Affiliations:** 1 Faculty of Physics, University of Tabriz, Tabriz, Iran; 2 Research Institute for Applied Physics and Astronomy (RIAPA), University of Tabriz, Tabriz, Iran; 3 Photonics Center of Excellence, University of Tabriz, Tabriz, Iran; 4 UNAM - Institute of Materials Science and Nanotechnology, Bilkent University, 06800 Ankara, Turkey

**Keywords:** Solar cells, Photovoltaics, Devices for energy harvesting

## Abstract

Numerical simulation of an all-perovskite bilayer solar cell has been conducted by the SCAPS-1D. The presented structure employs MAPbI_3_ as a relatively wide bandgap (1.55 eV) top absorber and FA_0.5_MA_0.5_Pb_0.5_Sn_0.5_I_3_ as a narrow bandgap (1.25 eV) bottom absorber. The viability of the proposed design is accomplished in two steps. First, to validate this study, two inverted solar cells in standalone conditions are simulated and calibrated to fit previously reported state-of-the-art results. Second, both these devices are appraised for the bilayer configuration to boost their performances. Affecting parameters such as the thickness of perovskite absorbers, the work function of front and rear contacts, and the effect of temperature have been studied because solar cells are temperature-sensitive devices, and also carrier concentration and their mobility get overwhelmingly influenced as temperature increases. It is manifested that using bilayer structures could easily widen the absorption spectrum to the near-infrared region and significantly enhance the performance of the device which is mainly affected by the thickness of the FA_0.5_MA_0.5_Pb_0.5_Sn_0.5_I_3_ layer. Also, it has been found that the work function of the front contact has a prominent role with its optimal values being above 5 eV. Finally, the optimized inverted all-perovskite bilayer solar cell delivers a power conversion efficiency of 24.83%, fill factor of 79.4%, open circuit voltage of 0.9 V, and short circuit current density of 34.76 mA/cm^2^ at 275 K and a thickness of 100 nm and 600 nm for MAPbI_3_ and FA_0.5_MA_0.5_Pb_0.5_Sn_0.5_I_3_, respectively.

## Introduction

Perovskites are proven to be one of the best solar cell materials due to their high absorption coefficient, low exciton binding energy, high power conversion efficiency, long diffusion length for both carriers, tunable bandgap, high dielectric permittivity, high transparency, and low-cost commercialization costs^1–3^. Perovskite solar cells (PSCs) have greatly improved in efficiency and have undergone significant design and manufacturing advances. This competency is established by their soaring power conversion efficiency since their first introduction in 2009 as solar cell absorbers reaching above 25% in 2022^4,5^. For single junction photovoltaic cells, a bandgap greater than 1.7 eV is not conducive^6,7^, so using a relatively wide bandgap is common. Also, although CsPbI_3_-based as a wide bandgap semiconductor has long-term stability and high V_oc_, these cells compared with MAPbI_3_ (relatively wide bandgap) have poor photoluminescence properties because of phase transitions. On the whole, one customary method to take advantage of photons with lower energy than bandgap is the use of multi-junction structural design^8^. Transparency properties of perovskite materials let researchers introduce multi-junction and bilayer (or multilayer heterojunction) structures for PSCs. Recently, a perovskite/Si tandem solar cell reached 32.5% efficiency and beat the SQ limit for a single junction solar cell^9^. Perovskites-based tandem solar cells have received tremendous attention in the construction of solar cells such as Sheng *et al.*^10^ proposed a monolithic all-perovskite tandem solar cell employing a solution-processed interlayer by using MAPbI_3_ and MAPbBr_3_ as the top and bottom sub-cells, respectively. Moghadamzadeh *et al.* ^11^ reported a 4 T all-perovskite tandem solar cell with a PCE of 23.6% and a stabilized PCE of 23.0%. Meng *et al.*^12^ studied the best partner material for CsPbI_3_, in which they proposed using CIGS with 1.1 eV bandgap as the bottom sub-cell and achieved 25.58% PCE for a 2-terminal tandem solar cell in simulations. Pandey *et al.*^13^ applied MAPbI_3_ for the top sub-cell and Si for the bottom sub-cell with thicknesses of 0.5 µm and 300 µm, respectively. Their simulation depicts PCEs around 27.6% for the 4-terminal tandem. Besides bilayer structures are constructed of wide bandgap (or relatively wide bandgap) and narrow bandgap stacked on top of each other to improve the PCE toward the Shockley-Queisser limit and broaden the absorption spectrum of single-junction solar cells. There are lots of perovskite semiconductor materials that can be replaced in bilayer structures because of their tunable band gaps. For instance, Khatoon *et al.*^14^ proposed a bilayer solar cell and found that CsPbI_3_/MAPbI_3_ bilayer perovskite solar cell efficiency is roughly double the efficiency of single junction CsPbI_3_ or MAPbI_3_ PSC. Zhang *et al.* ^15^ designed the CsPbI_x_Br_3−x_ /FAPbI_y_Br_3−y_ multi-hetero-junction perovskite solar cells with optimized energy band alignment, and achieve 17.48% PCE and excellent stability simultaneously. Ullah *et al.*^16^ by building a heterojunction bilayer absorption scenario of CsPbIBr_2_/CsPbBr_3_ achieved 15.89% efficiency. MAPbI_3_/MAPbI_2_Br cascade layers with 25.32% PCE were proposed by Lin *et al.*^17^ in which a novel energy band structure was used. Li *et al.*^6^ proposed a quantum dot inverted bilayer solar cell with a structure of α-CsPbI_3_/FAPbI_3_. They use FAPbI_3_ to broaden the absorbance spectrum and improved the efficiency from 12.3% for CsPbI_3_ to 15.6% for the bilayer device and also the stability of the device was improved as well. Akhtarianfar *et al.*^18^ believed that perovskite heterojunctions consisting of two different perovskite layers could make far-reaching changes in solar cell efficiency and stability and achieved high performance for CsPbI_3_/XPbI_3_ (X = FA or MA). CH_3_NH_3_PbI_3_/CsSnI_3_ heterojunction was investigated as a light-harvester by Duan *et al.*^19^ and the results disclosed that CsSnI_3_ makes wider the absorption spectrum that the narrow band gap CsSnI_3_ broadens the absorption spectrum to the near-infrared region and the high hole mobility favors efficient hole transfer and CsSnI_3_/CH_3_NH_3_PbI_3_ heterojunction has better photoelectric properties and PCE than CH_3_NH_3_PbI_3_ absorber layer. Xu *et al.*^20^ proposed an all-inorganic perovskite heterojunction with CsPbI_3_/CsSnI_3_ as the absorber and HTL-free design. Clark *et al.*^21^ developed a unique procedure to experimentally fabricate a series heterojunction in which APbX_3_/MASnX_3_ are perovskite layers, which showed substantial improvement in device performance. Wu *et al.*^22^ have systematically reviewed the evolution of PVSCs from single junction, heterojunction to multijunction designs. Furthermore, to break Shockley–Queisser (S–Q) limit for a single-junction solar cell, there are some structures (as mentioned before). One simple structure is to directly deposit one absorber on top of the other absorber to form a heterojunction. They showed that the modified heterojunction designs through perovskite/perovskite heterojunctions, perovskite/functional layer heterojunctions, and perovskite/organic BHJ heterojunctions enable achieving PCEs beyond 25%. Because the perovskite layer and the organic BHJ layer have complementary light absorption, this type of hybrid solar cell can not only reduce spectral losses but also save fabrication costs compared to typical tandem cells. Hence, organic solar cells^23^ because of their narrow bandgap can be used in both heterojunction and tandem solar cells. According to their work^22^, for double-absorber solar cells, three factors have to be noticed before the fabrication process: (i) good energy bands alignment between the two absorbers layers to achieve high solar cell performance, (ii) using low-cost raw materials for commercial purpose, (iii) being toxic-free for environmental reasons.

One effective method to avoid the disadvantage of single junction PVSCs is to construct bilayer or multilayer heterojunctions, which contain a series of different perovskite materials that absorb various regions of the light spectrum. As a result, a wider energy range of spectrum from the sunlight can be extracted and the generation of photogenerated carriers improves and then the PCE rises. There are limited studies on the impact of using double absorber layers compared to single absorber layers on solar cell performance ^24^. Here, some references have been investigated to compare the performance between single-junction and bilayer heterojunction solar cells. For instance, solar cells with a 3D perovskite layer as an active layer can be controlled through deposition techniques and crystallization modification of perovskite films, but these strategies have a nominal effect on the heterojunction properties. Perovskite/perovskite heterojunctions are constructed to alter the charge carrier dynamics between the perovskite and charge transporting layers. The employment of perovskite/perovskite heterojunctions can tune the energy level alignment and defect density at interfaces, thus affecting the device performance^22^. Carbon nitride (C_2_N), a graphene-like structure with extraordinary characteristics, is one of the interesting two-dimensional photovoltaic materials^25^. Ahmad *et al.* ^26^ presented the detailed numerical simulations of C_2_N-based solar cells with the (AI/TCO/Zn_1−x_ Mg_x_O/C_2_N)/Ni) device structure using the SCAPS-1D software. Yasin *et al.*^24^ reported on the optoelectronic simulation results of a new solar cell structure based on C_2_N/FASnI_3_ as double-absorber layers in the solar cell structure using SCAPS. Their results indicated that implementing double absorbers in the solar cell is more beneficial than a single absorber layer in terms of its efficiency. Finding the best partner junction for MAPbI_3_/IV-VI group semiconductor in bilayer design has been done by Hou *et al.*^27^ and they found that GeSe is the best partner among SnSe, GeS, and SnS. It turns out from their results that the bandgaps of these four materials are suitable for the absorption of long wavelength light (can absorb infrared light), which might have a complementary contribution to the perovskite that is almost predominant in the visible light, and the short circuit current density is enhanced 100% and the power conversion efficiency is promoted 42.7% (to a high value of 23.77%) larger than that in a solar cell with only single perovskite layer. Mohanty *et al.*^28^ proposed the perovskite/CIGS bilayer solar cell by studying on defect density of absorbers, and the mid-interface between absorbers obtained a high 28.15 efficiency for that structure. Their results showed that decreasing the defects in the absorber layer and improving the material quality will result in better device performance. AlZoubi *et al.* ^29^ proposed a new solar cell with the structure of ZnO:Al/CdS/CZTS/Si/Mo (CZTS/Si hybrid as double-absorber) and indicated that the combination of both CZTS and Si (adding a thin Si layer to the cell structure) forming a double absorber layer improves the light absorption by harvesting a wider range of visible light, which enhances the device performance. Heriche *et al.*^30^ added a p-type Si layer to ZnO/CdS/CIGS structure and enhanced the performance of the device from 16.39 to 21.3%. Their findings showed that the increase of the absorber layer thickness leads to the number of absorbed photons and the improvement of the performance of the bilayer solar cell. Prasad *et al.*^31^ performed a numerical simulation of a single absorber layer CuIn_0.69_Ga_0.31_Se_2_ solar cell device and bilayer CuIn_0.69_Ga_0.31_Se_2_/CuIn_0.55_Ga_0.45_Se_2_ solar cell under the illumination of AM 1.5 G.S. They simulated to study the influence on bilayer device (J–V) characteristics. They found that using a bilayer structure shows an increase in the device efficiency from 20.56 to 23.60% as compared with the single-layer cell. their result shows that the addition of an extra CIGS layer with a different composition leads to forming a higher efficiency device with improved output (J–V) characteristics.

Farooq *et al.*^32^ used a special way to improve solar cell performance. They numerically investigated the novel geometry of solar cells which is based on perovskite material as a photoactive layer and Si/SiO_2_ as a back reflector in DBR pairs. Their results provided a deep insight into the geometry. Their work aimed to achieve high conversion efficiency from perovskite solar cells by using a different number of pairs in DBR stacked. They investigated three different cases and found that the geometry of the cell based on CH_3_NH_3_PbBr_3_ with four pairs of DBR is super-efficient as compared to other geometries.

Additionally, much of the research is about regular perovskite structures while inverted perovskite solar cells have great spectacular specifics such as better stability than the other one. Furthermore, improvements in the efficiency of perovskite solar cells will require a better understanding of the roles played by each component and how they impact the photovoltaic device's performance, so the optimization of bilayer and tandem PCSs by modeling and numerical simulation helps researchers understand the basic physical processes in the experiment. The reason why the inverted p-i-n PVSCs (substrate/HTL/perovskite/ETL/top electrode) have received numerous attention from researchers is their simple device fabrication process, high PCE, and low hysteresis. The fabrication of p-i-n devices is desirable to suppress hysteresis due to the compensation of relatively shorter hole-diffusion length in the perovskite layer when compared to electron-diffusion length. Moreover, they have high environmental stability when compared to the n–i–p structure^33^. For inverted PSCs, the types of HTL can be divided into conductive—polymer HTL, organic small—molecule HTL, and inorganic—semiconductor HTL according to the property of materials^34^. In inverted PVSCs, PTAA, PEDOT:PSS, and NiO_x_ are the most popular materials for hole collection, and C_60_ and its derivatives (like PCBM) are the generally used electron collection materials that have good performances with doping-free systems which possessed excellent stability ^33^. PEDOT:PSS, as conductive − polymer materials, is used in this work because of its excellent electron conductivity, high transmittance, matched energy level, and low-temperature annealing process. Although, PEDOT: PSS has been extensively explored as HTL for inverted devices, PEDOT: PSS—based inverted PSC suffered a poor V_oc_^34^. In perovskite films, the often observed behavior is ion migration which is the main origin of the hysteresis phenomenon. Using inverted PVCs is one of the ways to suppress hysteresis effects. Ion migration can be limited if we use C_60_ or its derivatives like PCBM. Thus a key to suppressing the trap states and tying up the mobile ions to form a radical is the implementation of PCBM^2,35^.

In this article, the benefits and advantages of inverted all-perovskite bilayer solar cells combined with device optimization were investigated to achieve high efficiency for the bilayer PCS. MAPbI_3_ and FA_0.5_MA_0.5_Pb_0.5_Sn_0.5_I_3_ are used as top and bottom absorbers in the form of inverted structures in the studied bilayer device respectively. Numerical simulation shows a power conversion efficiency of 24.83% after all optimizations, say absorber layer, work-function of front and back electrode, and the working temperature. It cannot be neglected that, in regular (n–i–p) or inverted (p–i–n) planar PSCs, the absorbing layer is sandwiched between the electron transport layer (ETM) and hole transport layer (HTL)^36,37^, which in this paper PCBM, PEDOT: PSS are used as ETL and HTL in the inverted structure respectively.

## Methodology and simulation

**Figure 1:**
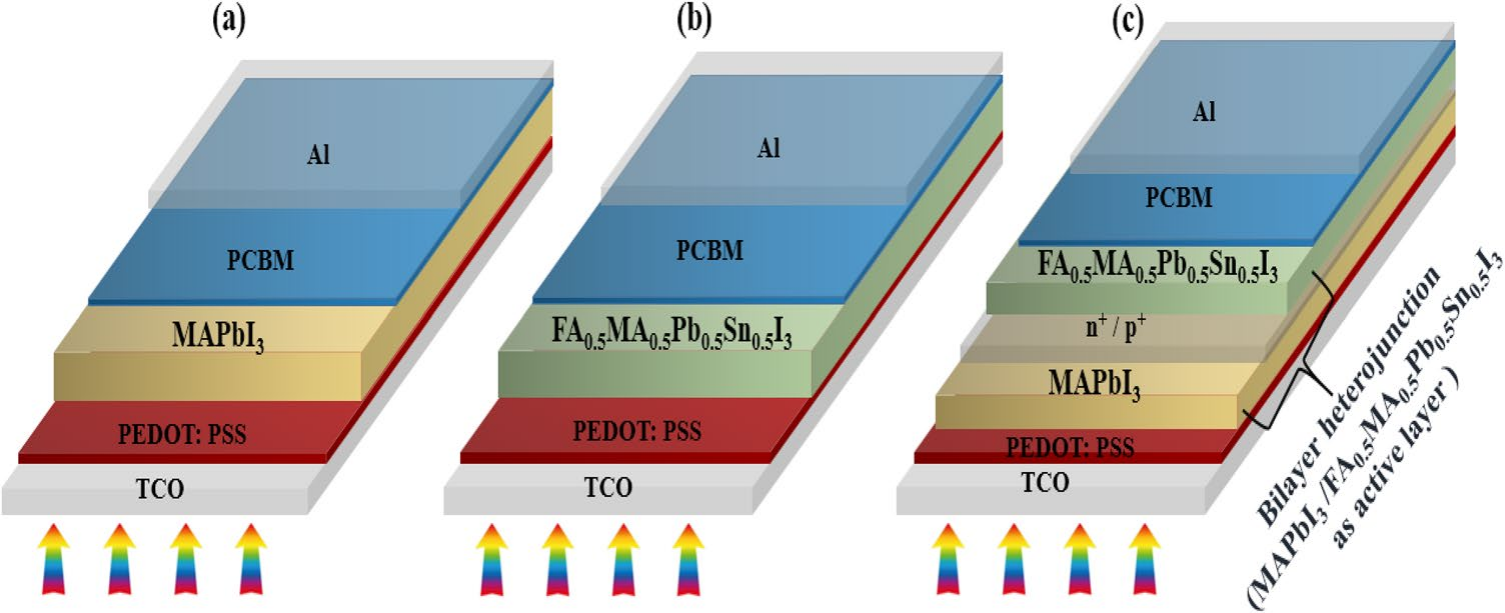
Simulated inverted structure of (**a**) MAPbI_3_-based perovskite solar cell. (**b**) FA_0.5_MA_0.5_Pb_0.5_Sn_0.5_I_3_ based perovskite solar cell. (**c**) Bilayer solar cell based on all-perovskite solar cell in which MAPbI_3_/ FA_0.5_MA_0.5_Pb_0.5_Sn_0.5_I_3_ acts as active layer.

The structure of the bilayer and perovskite junction solar cells has been shown in Fig. 1, where a thin layer of glass is placed at the front surface to maximize light absorption. To comprehend how the bilayer solar cell works, firstly two inverted single-junction solar cells with the same structure and different absorbent layers are simulated, and then the bilayer structure consisting of these two absorbent layers is evaluated. Figure 1a,b illustrate inverted single-junction solar cells with a perovskite layer as a light harvester also the structure of the inverted bilayer solar cell in this work consist of TCO/HTL/absorbent layer 1/absorbent layer 2/ETL/back contact is shown in Fig. 1c. Details of device simulation and numerical method are presented in supporting information. Simulation is used to assess the performance of an existing system, test ideas, and make predictions about a planned device. It cannot be neglected that using numerical simulation allows researchers to take a closer look at complex and large practical programs which is hard to solve through a mathematical method. In this work, to study the optoelectronic performance of the device and analyze data, SCAPS-1D is utilized. Besides, the simulations were carried out using wxAMPS (version 3.0) for validating the results attained from the SCAPS software. Fig. S7a,b indicate the comparison in J–V and QE curves between the two simulation tools. Furthermore, Table S2 has been provided to compare the values of the photovoltaic properties.

## Result and discussion

In this section, the bilayer solar cell consisting of the mentioned materials is examined and divided into 3 subsections. Section “Effect of thickness of top and bottom absorber layers” demonstrates the effect of absorber layers on bilayer characteristics. The performance of bilayer cells by varying front and rear contacts is discussed in Section “Effect of metal work-function of front and back contact”. In Section “Effect of temperature”, the efficacy of temperature on the bilayer cell is presented.

Before we start analyzing the performance of the bilayer solar cell, two inverted perovskite solar cells are investigated in standalone conditions, and the influence of thickness variation of absorber layers is studied. Also to validate our simulation, it has been compared with a similar case used in the experimental work done in Ref.^38,39^. The simulation results obtained in our work have a good agreement with the experimental data. The initial parameters for the materials used in the bilayer are listed in Table 1 and selected from the reported literature^40–44^.

**Table 1:** Employed simulation parameters.

Parameters	PEDOT:PSS^40^	PCBM^41^	MAPbI_3_^42^	FA_0.5_MA_0.5_Pb_0.5_Sn_0.5_I_3_^43^	TCO^40^
Thickness (nm)	40	20	100–800	100–800	500
Bandgap (eV)	1.55	2	1.55	1.25	3.5
Electron affinity (eV)	3.63	4.13	3.9	4.15	4
Dielectric permittivity	3	4	70 ^44^	8.2	9
Conduction Band Density of States (N_c_)	1 × 10^19^	1 × 10^19^	2.2 × 10^18^	1 × 10^19^	1 × 10^19^
Valance Band Density of States (N_v_)	1 × 10^19^	1 × 10^19^	1.8 × 10^19^	1 × 10^19^	1 × 10^19^
Electron/Hole mobility	9 × 10^–3^/9 × 10^–3^	1 × 10^–2^/1 × 10^–2^	2/2	2/2	2/1
Shallow uniform donor density (N_D)_	0	5 × 10^17^	1 × 10^15^	1 × 10^13^	2 × 10^19^
Shallow uniform acceptor density (N_A_)	3 × 10^17^	0	1 × 10^15^	0	1 × 10^15^

In this paper, MAPbI_3_, and FA_0.5_MA_0.5_Pb_0.5_Sn_0.5_I_3_ perovskites are considered as two absorber layers, which have 1.55 eV and 1.25 eV bandgaps, respectively. Table 2 provides the parameters for the Perovskite/ETM and Perovskite/HTM interfaces.

**Table 2:** Parameters taken for interface defects.

Parameters	HTM/Perovskite	ETM/Perovskite
Defect type	Neutral	Neutral
Cross-section area of electrons (cm^-2^)	1.2 × 10^–14^	1.2 × 10^–14^
Cross-section area of holes (cm^-2^)	1.2 × 10^–14^	1.2 × 10^–14^
Energetic distribution	single	single
Reference for defect energy level E_t_	Above the highest E_V_	Above the highest E_V_
energy from reference (eV)	0.6	0.6
Total density (cm^-3^)	1 × 10^9^	1 × 10^9^

**Figure 2:**
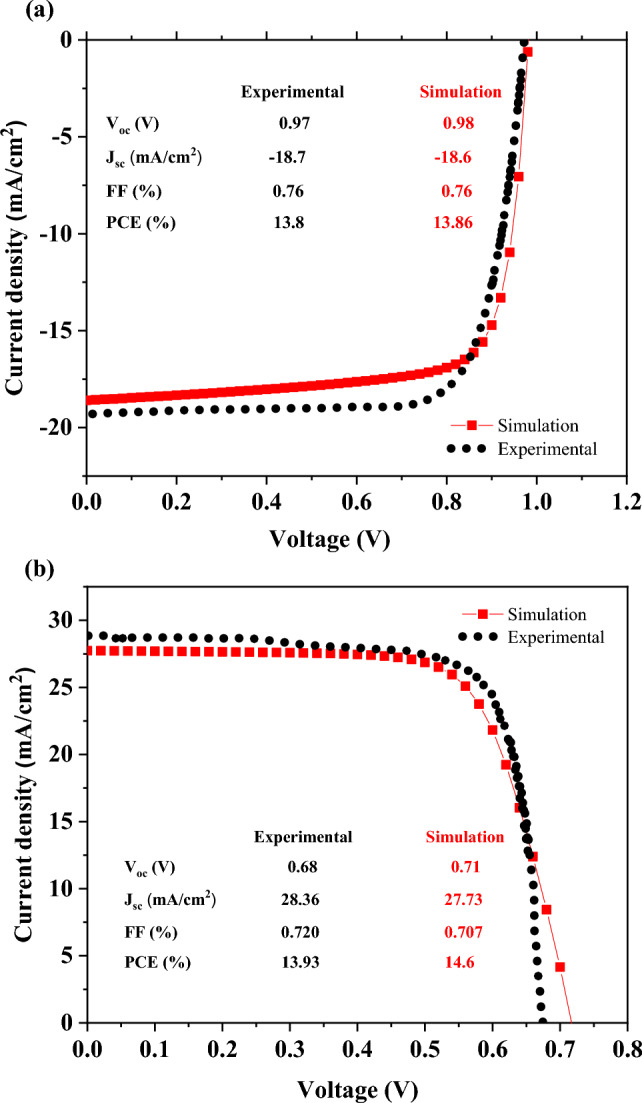
Comparison between simulation (red square) and reported experimental (dots) J–V characteristics of the (**a**) MAPbI_3_^38^_._ (**b**) FA_0.5_MA_0.5_Pb_0.5_Sn_0.5_I_3_^39^.

**Figure 3:**
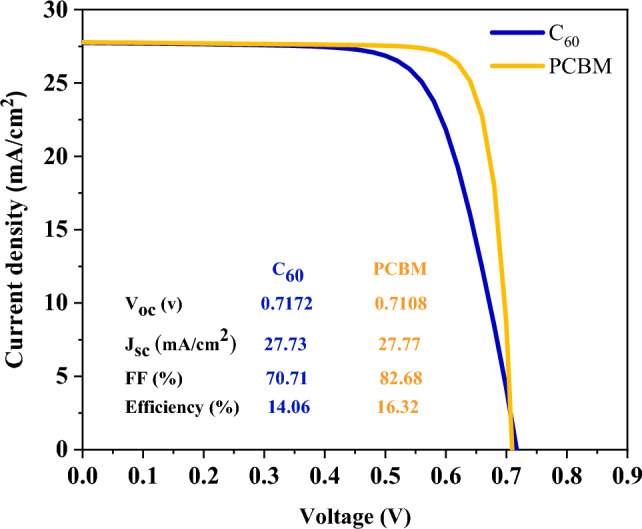
J–V curve of different ETL in FA_0.5_MA_0.5_Pb_0.5_Sn_0.5_I_3_ Perovskite-based solar cell.

Figure 2a,b demonstrate a comparison of the simulation and experimental work for solar cells with MAPbI_3_ and FA_0.5_MA_0.5_Pb_0.5_Sn_0.5_I_3_ absorption layers, respectively. In the bilayer architecture, to enable both the cells to have the same design, the electron transport layer, C_60_, have to be replaced by PCBM. It is clear from Fig. 3 that replacing C_60_ with PCBM improves the performance of the cell due to its higher mobility and fewer series resistance of PCBM than C_60_. The other reason could be the band offset between FA_0.5_MA_0.5_Pb_0.5_Sn_0.5_I_3_ and ETL (the band offset between FA_0.5_MA_0.5_Pb_0.5_Sn_0.5_I_3_ and PCBM is more suitable than that with C_60_).

**Figure 4:**
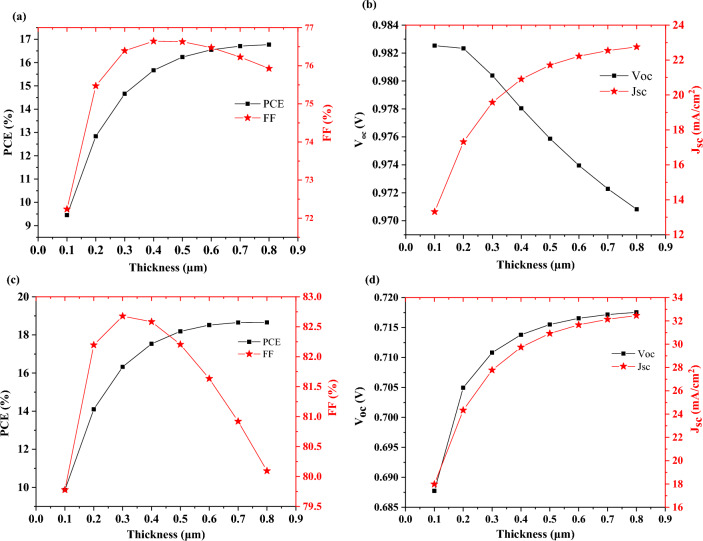
Effect of thickness of absorber layer on solar cell’s parameters (**a**, **b**) MAPbI_3_ and (**c**, **d**) FA_0.5_MA_0.5_Pb_0.5_Sn_0.5_I_3_.

Figure 4a,b display the thickness effect of the absorber layer on the performance parameters of a single-junction solar cell, as the thickness of the MAPbI_3_ layer increases, the efficiency and current density increase exponentially due to higher absorption. It is acquired that the thickness of the absorber layer (active layer) significantly increases the current density for both devices. The improvement can be attributed to optical absorption in the active region of the device and leads to excess electron–hole pairs. Open-circuit voltage decreases because of the reduction of Fermi level differences in the equilibrium state. Figure 4b shows that V_oc_ decreases from ~ 0.982 to ~ 0.972 V, after the thickness of 200 nm. This small decrease can be related to the bandgap of MAPbI_3_ which affects the built-in potential and consequently V_oc_. So to speak, collection efficiency for a thicker cell is low due to recombination which affects V_oc_. A decline in V_oc_ versus absorber thickness can be correlated to a decrease in the effective band gap with absorber thickness and an increase in recombination with thickness. As a result, a drop in V_oc_ might be due to high recombination.

The fill factor initially has an upward trend with increasing the thickness of the absorber layer, but in higher thicknesses of about 400 nm, it starts to decrease due to the domination of the recombination process and reduced carrier extraction. Also, as shown in Fig. 4c,d with increasing FA_0.5_MA_0.5_Pb_0.5_Sn_0.5_I_3_ thickness, the efficiency, current density, and open-circuit voltage increase exponentially, but the fill factor decreases at thicknesses higher than 300 nm. In contradiction to Fig. 4b,d indicates that V_oc_ increases from ~ 0.687 V to ~ 0.717 V. The reason why Fig. 4d does not have the same trend as Fig. 4b is the smaller bandgap of FA_0.5_MA_0.5_Pb_0.5_Sn_0.5_I_3_ (compared to MAPbI_3_) and good band alignment with PEDOT: PSS and PCBM as HTL and ETL, respectively.

The initial simulation of the bilayer cell with parameters in Table 1 was performed under AM 1.5 standard light irradiation and radiation power of 1000 W/m^2^. An efficiency of 19.79%, fill factor of 77.67%, current density of 28.81 mA/cm^2^, and open-circuit voltage of around 0.88 V was obtained for the bilayer cell in the initial simulation (see Fig. S1 in supporting information). By varying the parameters of the absorber layers and work-function of the contacts we show improved performance for the proposed device. In addition, it is illustrated the temperature of the cell plays a significant role in the functionality of this bilayer cell.

### Effect of thickness of top and bottom absorber layers

To optimize the performance of the proposed bilayer cell, firstly the thickness of the perovskite layers varies from 100 to 800 nm. Unlike the previous analyses, an increase in the thickness of MAPbI_3_ would adversely affect the performance of the bilayer cell. This is mainly due to the high absorption of the MAPbI_3_ layer and the bandgap of this layer that is close to the bottom absorber's bandgap. As the thickness of the top absorber increase, the number of transmitted photons that reach to bottom absorber layer decreases. Thus, the thickness of this layer should be chosen as thin. Also, using a thin layer of MAPbI_3_ can constructively mitigate the thermionic emission due to its relatively wide bandgap in the bottom absorber.

**Figure 5:**
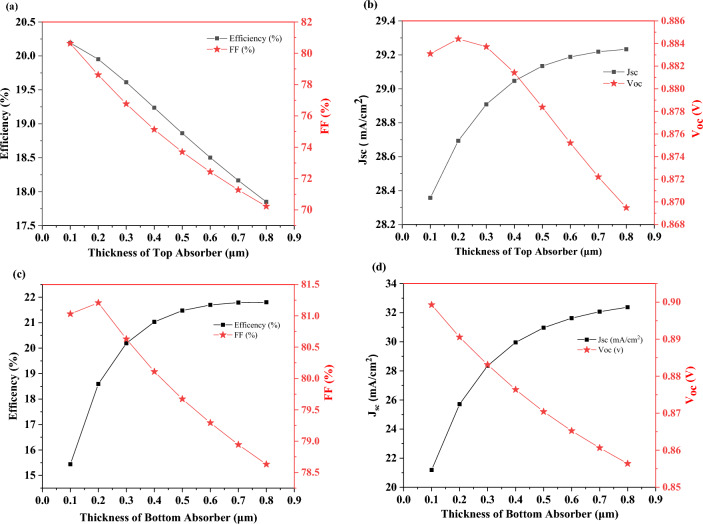
Effect of thickness of top (**a**, **b**) and bottom absorber (**c**, **d**) on bilayer characteristic.

Figure 5a–d indicates the thickness effect of perovskite layers on the photovoltaic parameters.

According to equation (S1) in supporting information, the increase in the dark saturation current (J_0_) is the cause of the reduction in open circuit voltage (V_oc_), and the increase in short circuit current density (J_sc_) is basically due to high photon absorption and enhancement in carrier generation (see Fig. 5b,d). On the one hand, a thicker perovskite layer absorbs more sunlight and produces more photocarriers, so as the thickness of absorber layer increases, the J_sc_ increases and then tends to saturation. Meanwhile, in a thicker absorber layer, the charges have to travel longer distances for diffusion, so the chance of recombination increases. On the other hand, the V_oc_ reduces because as the thickness of absorber layer increases, the carrier diffusion length rises and the recombination rate of carriers increases.

As the thickness of the MAPbI_3_ layer increases, the fill factor decreases (see Fig. 5a). This issue can be attributed to the overall resistance of the device. It can be concluded from the results that in contrast to the other characteristics of solar cells (V_oc_, J_sc_, and FF) which have almost the same trend, the increase in the thickness of two absorbers has different trends for PCE, as shown in Fig. 5a,c.

This is because as the thickness of a relatively wide bandgap layer increases, the absorption will increase, and hence the performance of device will degrade. Conversely, for the absorber layer with a narrow-bandgap increase in thickness will cause better efficiency because some parts of sunlight with lower wavelength are absorbed by absorber layer with wide-bandgap. As mentioned above, the thickness of these two layers has a significant effect on the performance of the bilayer cell and the thickness of the wide bandgap absorber layer should be thinner than the narrow bandgap.

**Figure 6:**
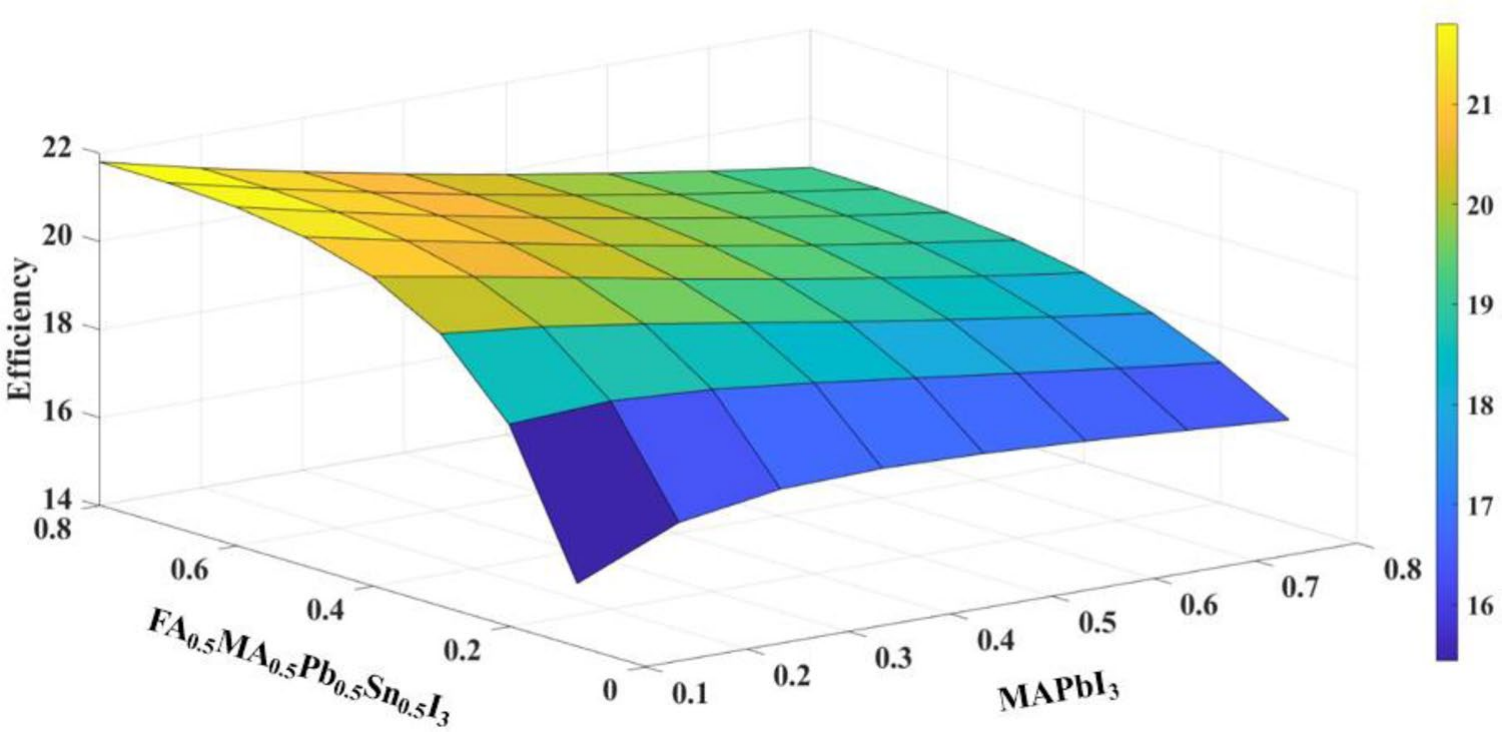
The 3D plot of inverted all-perovskite bilayer.

To find optimized thickness for two absorber layers, it is essential to use a 3D plot (Fig. 6). It is obvious from Fig. 6 that the best thickness for MAPbI_3_ and FA_0.5_MA_0.5_Pb_0.5_Sn_0.5_I_3_ is 100 nm and 600 nm respectively.

The ratio of the number of carriers collected by the solar cell to the number of incident photons is called the quantum efficiency of a solar cell. It describes the response of the device to different wavelengths of light. It represents the ratio of the number of collected carriers in short-circuit operating conditions by the number of incident photons for a given wavelength.

**Figure 7:**
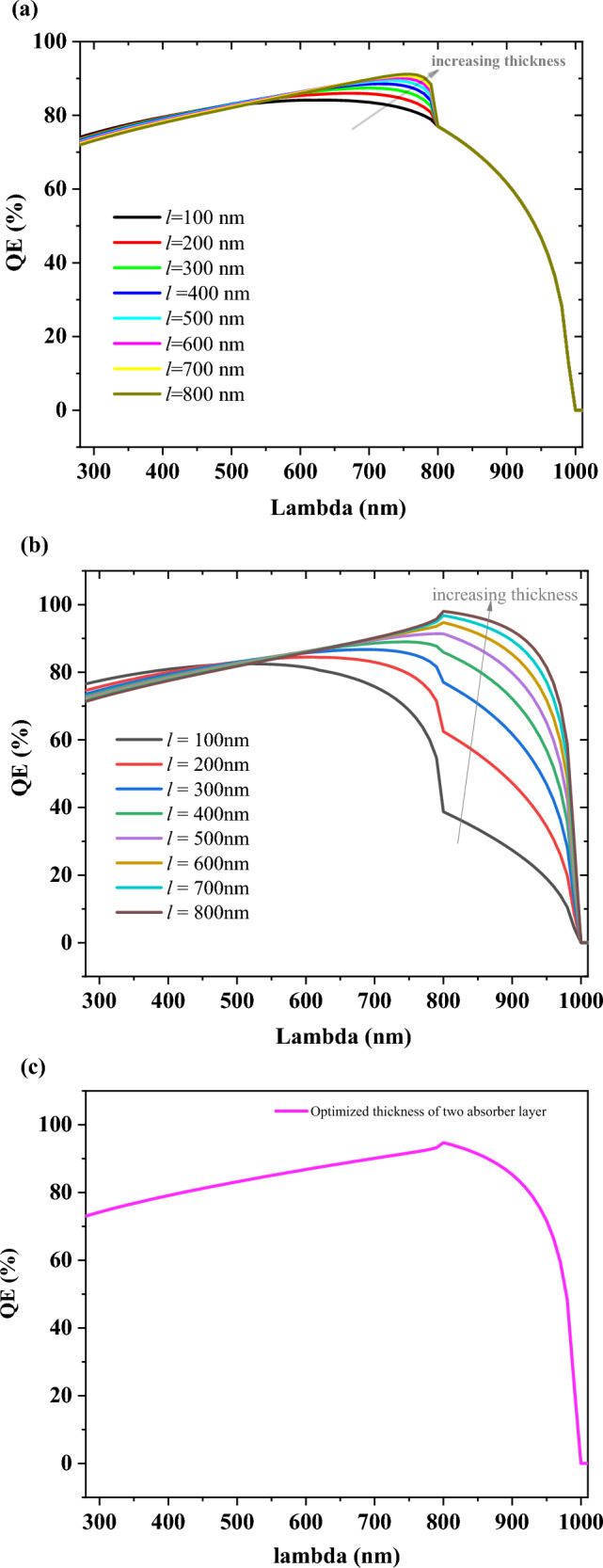
Analysis of Quantum Efficiency, (**a**) impact of varying of top absorber (MAPbI_3_) from 100 to 800 nm, (**b**) impact of varying of bottom absorber (FA_0.5_MA_0.5_Pb_0.5_Sn_0.5_I_3_) from 100 to 800 nm, (**c**) optimized thickness in bilayer structure: at the thickness of 100 nm and 600 nm for top absorber and bottom absorber, respectively and at 300 K.

It is interpreted from Fig. 7a,b that the effect of the thickness of MAPbI_3_ in the bilayer structure is less than FA_0.5_MA_0.5_Pb_0.5_Sn_0.5_I_3_. EQE for both absorbing layers is improved due to an increase in J_sc_, but an increase in the thickness of MAPbI_3_ is not salient. So, it is obtained that FA_0.5_MA_0.5_Pb_0.5_Sn_0.5_I_3_ as an active layer plays a crucial role in the proposed bilayer device. Finally, in Fig. 7c, EQE for optimized thickness has been shown.

### Effect of metal work-function of front and back contact

**Figure 8:**
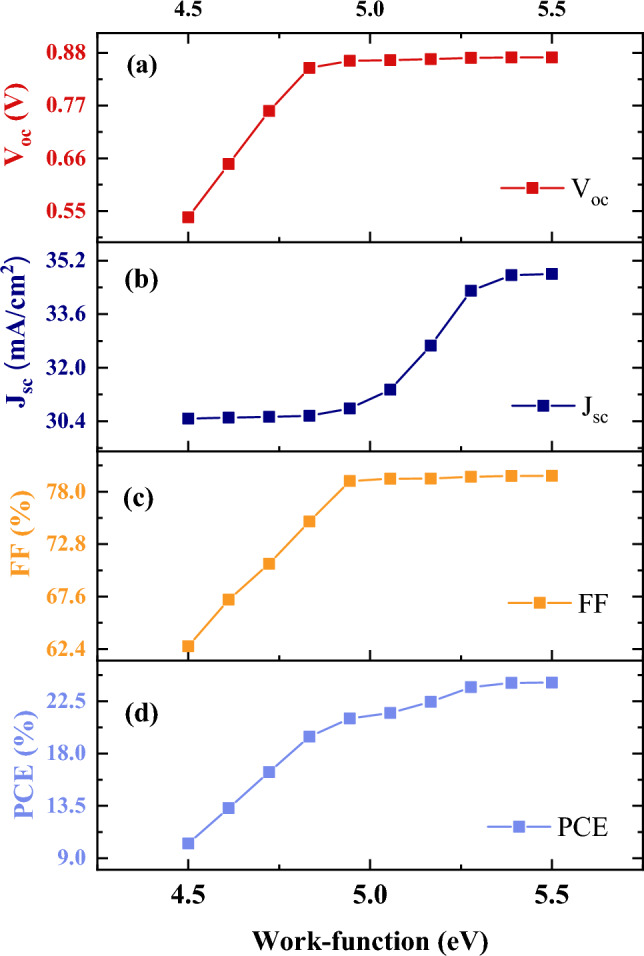
Effect of work-function of front contact on the bilayer performance.

In addition to the thickness of the absorbers, the work function of the front and rear electrodes play an essential role in improving or diminishing the performance of the bilayer cell. Figure 8 shows the effects of electrode work function on the bilayer cell parameters. As shown in this figure, changing the work function of the front electrode from 4.5 to 5.5 eV leads to efficiency variations from 10.25 to 24.10%.

Also, variations in the work function of the rear electrode from 2.0 to 4.2 eV result in the reduction of the efficiency (Fig. S3). It is concluded that the effect of the front electrode is higher than the rear electrode, so the main contact in our device is the front electrode because an increase in the work function of the front contact leads to an increase in PCE. It is clear from Fig. 8 that as the work function of the front electrode is varied from 4.5 to 5.5 eV, all the parameters of the solar cell increase.

To avoid a high Schottky barrier, promote the photogenerated carriers, and consequently increase the performance of a device, the work function of the front and rear contacts are set to be 5.4 eV and 4.2 eV, respectively.

Applying the band diagram can be useful for perusing the effect of work function on device performance.

**Figure 9:**
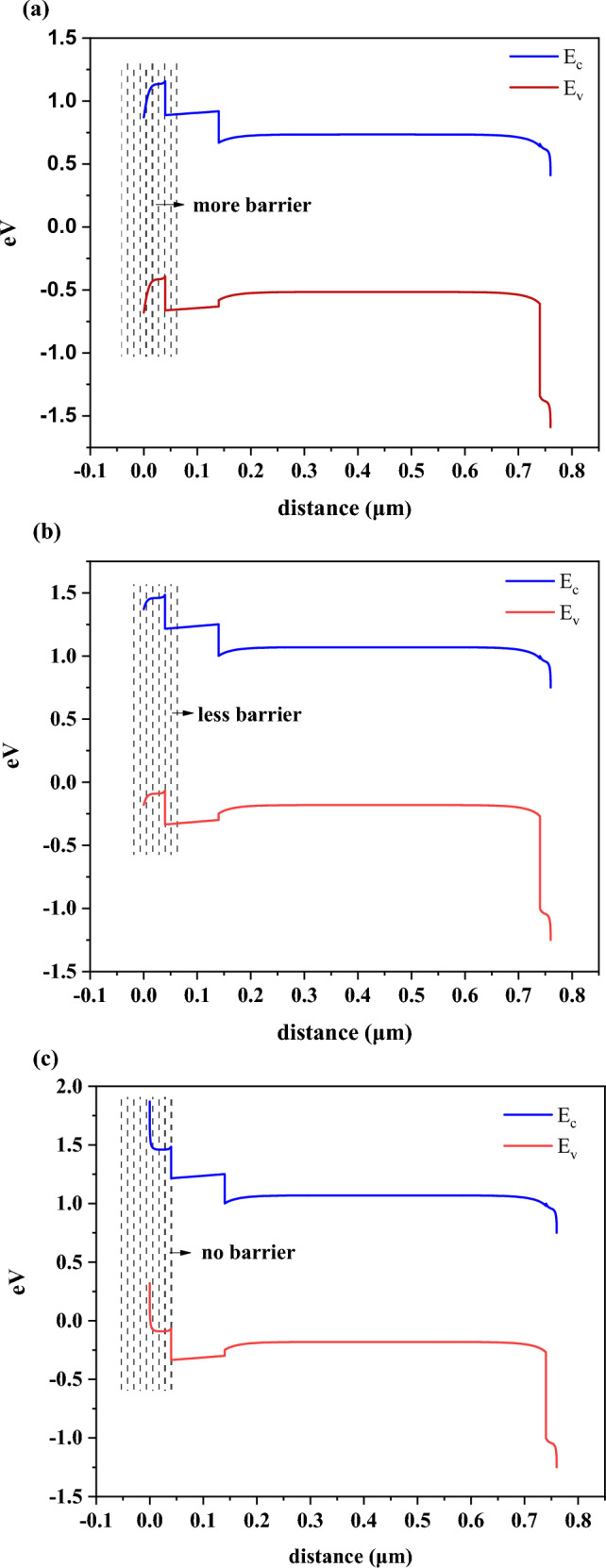
Effect of work-function of front contact on the band diagram at (**a**) 4.5 eV, (**b**) 5 eV, and (**c**) 5.5 eV.

When the work-function of the front electrode is 4.5 eV (Fig. 9a) there is a large barrier, and also increase in work-function will be subjected to less barrier (Fig. 9b) and no barrier in high work-function (Fig. 9c). It is elucidated from Fig. 9, the low work-function of the front electrode degrades the device's performance because of the large barrier against flowing carriers. On the other hand, as the front electrode metal work function increases up toward 5.5 eV, the performance of the device, together with solar cell parameters, improves.

### Effect of temperature

All the simulations were done while the temperature had been kept at 300 K. In this section, the temperature was changed from 275 to 425 K to take into account the influence of the working temperature on the PCE, V_oc_, J_sc_, and FF of solar cells. The temperature dependence of solar cell parameters is explained in supporting information.

**Figure 10:**
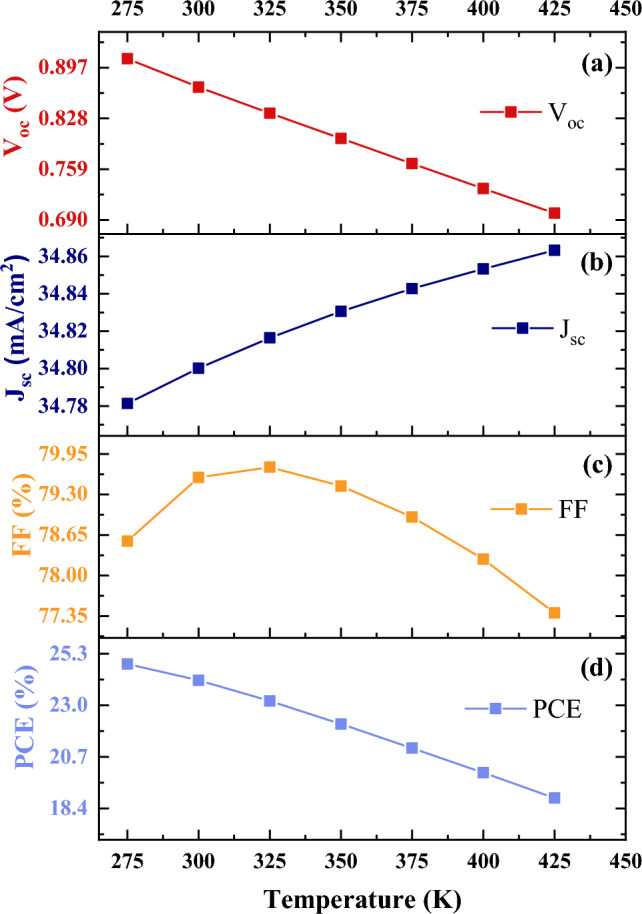
Effect of temperature on solar cells parameters, (**a**) open circuit voltage, (**b**) short circuit current, (**c**) fill Factor, (**d**) power conversion efficiency.

One of the reasons that the open-circuit voltage decreases sharply can be attributed to the fact that an increase in temperature leads to a reduction in E_g_ and eventually V_oc_, also according to equations (S8) and (S9), the other reason may be reverse saturation current density J_0_. J_0_ is a measure of recombination of minority across the p–n junction in reverse bias, so J_0_ has a key role in controlling the value of V_oc_ (Fig. 10a). The value of J_sc_ can be restricted by series and shunt resistance (ohmic losses), front metal coverage (shadowing losses), reflection loses, and recombination losses. In our simulation, the current density is constant with increasing temperature. (Fig. 10b). Figure 10c demonstrates that FF starts degrading after a certain value. This is mainly due to a reduction in V_oc_ and enhancement in series resistance.

Eventually, Fig. S4 shows that as the temperature varies from 275 to 425 K, constant J_sc_, and reduction in both V_oc_ and FF lead to a decrease in the PCE of the device (see Fig. 10d).

**Figure 11:**
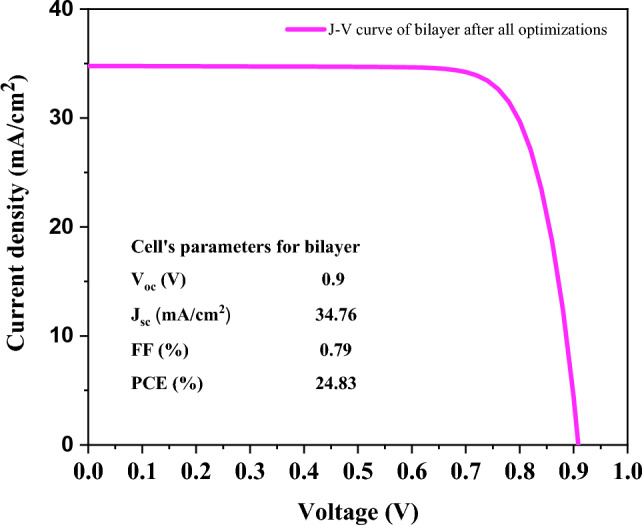
The J–V curve of bilayer after all optimizations (at 275 K, the thickness of MAPbI_3_ and FA_0.5_MA_0.5_Pb_0.5_Sn_0.5_I_3_ is 100 nm and 600 nm respectively, and work-function of front and back contact is 5.4 eV and 4.2 eV, respectively.).

It is found from the J–V curve (Fig. 11) after all optimizations PCE = 24.83%, Fill Factor = 79%, J_sc_ = 34.76 mA/cm^2^, V_oc_ = 0.9 V can be obtained from the bilayer.

Table 3 shows the differences between the parameters of the bilayer cell and the other two cells. It is found from the results that using two absorber layers in this work leads to an increase in PCE, FF, and J_sc_, and almost average of V_oc_, so using bilayer structures can be one of the methods to improve the solar cell’s performance.

**Table 3:** Compare solar cell parameters for used cells.

Cells/Parameters	PCE (%)	FF (%)	J_sc_ (mA/cm^2^)	V_oc_ (V)
MAPbI_3_	13.84	76	18.6	0.98
FA_0.5_MA_0.5_Pb_0.5_Sn_0.5_I_3_	14.6	70.7	27.73	0.71
bilayer (initial Simulation)	19.79	77.67	28.81	0.88

The results of recently published simulation and experimental studies on all-perovskite bilayer solar cells are summarized in Table 4 and selected from the reported literature^6,45–52^. Farhadi *et al.*^45^ and Abedini-Ahangarkola *et al.*^46^ attained over 30% PCE, and in both works, the same ETL (TiO_2_) and different HTL were used. To improve the performance of all-perovskite solar cells there are disparate tactics like deposition techniques, engineering of interfaces, charge recombination layers and additives, etc. For this perspective, Zhang *et al.*^15^ realized the multi-junction with a structure of C_60_/CsPbIxBr_3-x_/FAPbIyBr_3-y_ had high electron transportation and low carrier recombination rate and they acquired 17.48% (as mentioned above) for that device. They found a great relationship between structure, photoelectron, and photovoltaic properties which led to harvesting more solar light and simplifying the extraction of the electron. Furthermore, Ghahremanirad *et al.*^53^ proposed a double absorber solar cell and a new design in which a plasmonic network and kieserite mesoscopic lead to a broad absorbance spectrum. They found that the expectancy of light harvesting can be ameliorated by kieserite NPs which can absorb high wavelength (low energy). Our proposed device model achieves an efficiency of 24.83% compared with the published data. The same work is done by Singh *et al.*^47^ and remarkable photovoltaic parameters by analyzing the different ETL, perovskite layer thickness, and its defect density on photovoltaic performance and the effect of front electrode work function on device photovoltaic performance obtained. Their work showed that open circuit voltage (V_oc_) is significantly affected by the built-in voltage (V_bi_) across the perovskite layer.

**Table 4:** Simulated and fabricated results of all–perovskite bilayer solar cell.

Device structure	PCE (%)	FF(%)	J_sc_ (mA/cm^2^)	V_oc_ (V)	References
FTO/TiO_2_/ CsPbI_3_/ FAPbI_3_/PTAA/ MoO_3_/Ag (experimental)	15.60	74	17.26	1.22	^6^
FTO/TiO_2_/MASnI_3_/MAPbI_3_/Cu_2_O/Au	30.88	80.21	31.58	1.21	^45^
FTO/TiO_2_/MASnI_3_/ MAPbI_3_/Spiro-OMeTAD/Au	30.29	85.29	30.87	1.15	^46^
FTO/ZnO/CH_3_NH_3_GeI_3_/FAMASnGeI_3_/Cu_2_O/Au	26.72	84.46	28.36	1.07	^47^
ITO/TiO_2_/Cs_0.25_FA_0.75_PbI_3_/CsPbI_3_/Spiro-OMeTAD/MoO_x_/Al (experimental)	17.39	76	18.91	1.20	^48^
ZnO:Al/TiO_2_/MASnI_3_/MASnBr_3_/CuI/Au	24.78	68.82	32.03	1.12	^49^
FTO/WS_2_/MAPb(I_1-x_Cl_x_)_3_/FA_0.75_Cs_0.25_Pb_0.5_Sn_0.5_I_3_/Au	22.72	78.54	34.34	0.84	^50^
FTO/TiO_2_/CsPbI_3_/ MAPbI_3_/Spiro-OMeTAD/Au	20.99	74.24	25.02	1.13	^51^
FTO/TiO_2_/CsPbI_3_/ FAPbI_3_/Spiro-OMeTAD/Au	20.22	71.16	26.53	1.07	^51^
FTO/CdZnS/MASnI_3_/MASnBr_3_/rear contact	18.71	68.88	31.42	0.87	^52^
FTO/PEDOT:PSS/MAPbI_3_/ FA_0.5_MA_0.5_Pb_0.5_Sn_0.5_I_3_ PCBM/rear contact	24.83	79.4	34.76	0.9	This work

Taking a closer look at the comparison with the references, it turns out that using the bilayer structure is a great way to widen the light spectrum and increase the performance of solar cell devices. Also, it is illustrated in Table 4 that all simulation or experimental work is regular-based PSCs in contrast with our work.

## Conclusion

In this study, SCAPS-1D was employed to analyze the performance of an inverted all-perovskite bilayer solar cell with the structure of Fluorine doped tin oxide (FTO)/PEDOT: PSS/MAPbI_3_/FA_0.5_MA_0.5_Pb_0.5_Sn_0.5_I_3_/PCBM/back-contact. Until recently, there are few reports based on inverted all-perovskite bilayer solar cells. The present simulation results reveal that the combination of MAPbI_3_ and FA_0.5_MA_0.5_Pb_0.5_Sn_0.5_I_3_ could notably broaden the absorption spectrum of the cell and raise the J_sc_ and PCE. Optimization of the absorbing layer thickness lets us have high photon absorption without a high recombination rate. The optimized thickness of MAPbI_3_ and FA_0.5_MA_0.5_Pb_0.5_Sn_0.5_I_3_ was found to be 100 nm and 600 nm, respectively. Additionally, the defect density of MAPbI_3_ and FA_0.5_MA_0.5_Pb_0.5_Sn_0.5_I_3_ is set to be 10^14^ (1/cm^3^) and 2 × 10^13^ (1/cm^3^) respectively to have less excessive carrier recombination centering on the cell and promote the carrier lifetime. Moreover, it is obtained from the results that the front contact is the main contact, and to avoid the Schottky barrier, its value must not be less than 4.8 eV. The carrier recombination rate would increase in terms of enhancement in working temperature, which inhibits the output of V_oc_. After implementing the optimization, a power conversion efficiency (PCE) of 24.83%, fill factor (FF) of 79.4%, open circuit voltage (V_oc_) of 0.9 V, and short circuit current density (J_sc_) of 34.76 mA/cm^2^ are obtained. Comparing the results of the bilayer with individual structures shows preferable PV performance, thus our work helps to understand and improve the perovskite solar cells.

## Supplementary Information

Supplementary Information.

## Data Availability

The datasets used and/or analyzed during the current study are available from the corresponding author upon reasonable request.
