# Supplementary material for: Performance analyses of highly efficient inverted all-perovskite bilayer solar cell
Source: Sci Rep. 2023 May 22;13:8274. doi: 10.1038/s41598-023-35504-x (PMC10203109; doi:10.1038/s41598-023-35504-x)
Supplement: Supplementary file 1 — Supplementary Information. [file 41598_2023_35504_MOESM1_ESM.docx]

Supporting Information

to

**Performance analyses of highly efficient inverted all-perovskite bilayer solar cell**

Alireza Gholami-Milani^1,2^, Sohrab Ahmadi-Kandjani^1,2,3,*^, Babak Olyaeefar^4^, Mir Hojjat Kermani^1^

^1^ Faculty of Physics, University of Tabriz, Tabriz, Iran

^2^ Research Institute for Applied Physics and Astronomy (RIAPA), University of Tabriz, Tabriz, Iran

^3^ Photonics Center of Excellence, University of Tabriz, Tabriz, Iran

^4^ UNAM - National Nanotechnology Research Center, Bilkent University, 06800 Ankara, Turkey

* [s_ahmadi@tabrizu.ac.ir](mailto:s_ahmadi@tabrizu.ac.ir) or [ahmadi.sohrab@gmail.com](mailto:ahmadi.sohrab@gmail.com@tabrizu.ac.ir)

# The main parameters of the bilayer solar cell

Drift-diffusion equations that govern the performance examination of a typical solar cell are the Poisson equation, the coupling equation for electrons, and the coupling equation for holes. In general, the Poisson equation can be written as follows:

$\frac{d}{\mathrm{dx}}\left( -\varepsilon\left( x \right)\frac{d\Psi}{\mathrm{dx}} \right)= q\left[ p\left( x \right)-n\left( x \right)+N_{D}^{+}-N_{A}^{-}+p_{t}\left( x \right)-n_{t}\left( x \right) \right]$ **(S1)**

Where Ψ electrostatic is potential, n and p are free electron and hole densities, respectively. $n_{t}$ and $p_{t}$ are trapped electron and hole densities, respectively. $N_{D}^{+}$ and $N_{A}^{-}$ are the density of donor and acceptor carriers. ε is the semiconductor dielectric conductivity, and q is the electron charge. Properties of the carriers in this structure are derived from the continuum equation for electrons and holes in steady-state as:

$\frac{1}{q}\frac{\mathrm{dJn}}{\mathrm{dx}}=R_{n}\left( x \right)-G(x)$ **(S2)**

$\frac{1}{q}\frac{\mathrm{dJp}}{\mathrm{dx}}=G\left( x \right)-R_{p}\left( x \right)$ **(S3)**

In Eq.2 and Eq.3, J_n_ and J_p_ determine the current density of the electron and the hole. R_n_ and R_p_ represent recombination routes for electrons and holes. G (x) stands for carrier generation with an x spatial dependency. Simultaneous solving of the above-mentioned differential equations (Eq.1-3) with considering proper boundary conditions gives the density of the carriers, electric field distribution, and current inside the cell. We use SCAPS, a Drift Diffusion solving package developed by the University of Ghent to evaluate our bilayer design. This program has the capability of simulating up to seven distinct material layers. We extract the current-voltage (IV) curve, quantum efficiency (QE), as well as photovoltaic parameters: short-circuit current density (J_SC_), open-circuit voltage (V_oc_), filling factor (FF), and PCE.

The current density of the cell is determined by the voltage. In this case, the excitation of exaction and the transfer of charge are done by the internal potential of the cell. Also, the voltage per zero current is called the open circuit voltage, which can be calculated from Equation (S1):

$\mathrm{V}_{\mathrm{oc}}=\mathrm{nV}_{\mathrm{T}}\ln[\exp(\frac{\mathrm{I}_{\mathrm{sc}}}{\mathrm{I}_{0}})+1]$ **(S4)**

where J_0_ is the dark saturation current, n is the diode ideality factor, IG is the light-generated current and V_T_ is the thermal voltage.

Where I_0_ is the reverse saturation current, the ratio of maximum power to product of short-circuits current density at open-circuit voltage is called the charge factor, which can be calculated from Equation (S2):

$\begin{matrix} \mathrm{FF}=\frac{\mathrm{J}_{\mathrm{m}}\mathrm{V}_{\mathrm{m}}}{\mathrm{J}_{\mathrm{sc}}\mathrm{V}_{\mathrm{oc}}} \\ \mathrm{FF}=\frac{\mathrm{V}_{\mathrm{oc}}-\ln(\mathrm{V}_{\mathrm{oc}}+0.72)}{\mathrm{V}_{\mathrm{oc}}+1} \end{matrix}$ **(S5)**

Where P_m_ is the maximum power, the power conversion efficiency can also be calculated from Equation (S3):

$\mathrm{PCE}=\frac{\mathrm{P}_{\mathrm{m}}}{\mathrm{P}_{\mathrm{in}}}=\frac{\mathrm{V}_{\mathrm{oc}}.\mathrm{J}_{\mathrm{sc}}.\mathrm{FF}}{\mathrm{P}_{\mathrm{in}}}$ **(S6)**

Where P_in_ is the intensity of the incident radiation. This power is usually considered to be 1000 watts per square meter under irradiation AM1.5.

Photon production flux is obtained from [1,2]:

$I_{\mathrm{ph}}=q\int_{\lambda} \varphi\left( \lambda\right)\left\{ 1-R\left( \lambda\right) \right\}\mathrm{QE}\left( \lambda\right)d \lambda$ **(S7)**

Where QE (λ) is the quantum efficiency of the solar cell. Solving Eq.7 for photon production current is equivalent to the quantum efficiency, which means that as the quantum efficiency increases, the efficiency of the solar cell improves.

# Simulating the experimental FA_0.5_MA_0.5_Pb_0.5_Sn_0.5_I_3_ single-junction solar cell

**Table S1**: Employed simulation parameters for FA_0.5_MA_0.5_Pb_0.5_Sn_0.5_I_3_ single-junction solar cell.

| Parameters | PEDOT:PSS | C_60_ | FA_0.5_MA_0.5_Pb_0.5_Sn_0.5_I_3_ | TCO |
| --- | --- | --- | --- | --- |
| Thickness (nm) | 40 | 25 | 300 | 500 |
| Bandgap (eV) | 1.55 | 1.7 | 1.25 | 3.5 |
| Electron affinity (eV) | 3.63 | 4.5 | 4.15 | 4 |
| Dielectric permittivity | 3 | 18 | 8.2 | 9 |
| Conduction Band Density of States (N_c_) | 1 × 10^19^ | 1 × 10^19^ | 1 × 10^19^ | 1 × 10^19^ |
| Valance Band Density of States (N_v_) | 1 × 10^19^ | 1 × 10^19^ | 1 × 10^19^ | 1 × 10^19^ |
| Electron / Hole mobility | 9 × 10^-3^ / 9 × 10^-3^ | 8 × 10^-2^ / 8 × 10^-2^ | 2 / 2 | 2 / 1 |
| Shallow uniform donor density (N_D)_ | 0 | 1 × 10^18^ | 1 × 10^13^ | 2 × 10^19^ |
| Shallow uniform acceptor density (N_A_) | 3 × 10^17^ | 0 | 0 | 1 × 10^15^ |

# Initial simulation of bilayer cell

Fig. S1 shows the initial simulation of bilayer solar cell. An efficiency of 19.79%, fill factor of 77.67%, current density of 28.81 mA/cm^2^, and open-circuit voltage of around 0.88 V was obtained for the bilayer cell at the thickness of 250 nm and 300 nm for MAPbI_3_ and FA_0.5_MA_0.5_Pb_0.5_Sn_0.5_I_3_, respectively,

**Fig. S1:** (Color online) Initial simulation of bilayer cell.

# Spectrum

Fig. S2 shows the irradiation AM1.5 in comparison with AM1.5 filtered by MAPbI_3_. Also, the figure demonstrates how a 100 nm thickness of MAPbI_3_ can absorb the AM 1.5G.

**Fig. S2:** (Color online) AM 1.5G filtered by MAPbI_3_ with a thickness of 100 nm.

# Work-function of contacts

**Fig. S3:** (Color online) Effect of work-function of the front (a) and back (b) contact on the bilayer performance.

# Temperature

The temperature has a key role in studying solar cell parameters. The relation for variation of the energy gap (E_g_) versus temperature (T) in semiconductors (Varshni relation) can be described as:

 **(S7)**

where, Eg (T) is the band gap of the semiconductor at some temperature T, which may be direct or indirect, and Eg (0) is its value at T, and ‘a’ and ‘b’ are constants.

According to equation S1, the temperature dependence of V_oc_ can be obtained as:

$\begin{matrix} \frac{\mathrm{dV}_{\mathrm{oc}}}{\mathrm{dT}}=(\frac{\mathrm{V}_{\mathrm{oc}}}{\mathrm{T}})+\mathrm{V}_{\mathrm{T}}(\frac{1}{\mathrm{J}_{\mathrm{sc}}}\frac{\mathrm{dJ}_{\mathrm{sc}}}{\mathrm{dT}}-\frac{1}{\mathrm{J}_{0}}\frac{\mathrm{dJ}_{0}}{\mathrm{dT}}) \\ \mathrm{V}_{\mathrm{T}}=\frac{\mathrm{kT}}{\mathrm{q}} \end{matrix}$ **(S8)**

 **(S9)**

According to equation S2, the temperature dependence of FF can be obtained as:

$\frac{\mathrm{dFF}}{\mathrm{dT}}=(\frac{\frac{\mathrm{dV}_{\mathrm{oc}}}{\mathrm{dT}}-\frac{\mathrm{V}_{\mathrm{oc}}}{\mathrm{T}}}{\mathrm{V}_{\mathrm{oc}}+\mathrm{V}_{\mathrm{T}}})+\{\frac{(\frac{\mathrm{V}_{\mathrm{oc}}}{\mathrm{V}_{\mathrm{T}}}-0.28)}{(\frac{\mathrm{V}_{\mathrm{oc}}}{\mathrm{V}_{\mathrm{T}}}+0.72)}-\mathrm{FF}\}$ **(S10)**

Calculations have been done somewhere else [3].

Eventually, the effect of operating temperature on the J-V curve of the bilayer is shown in Fig. S4.

**Fig. S4:** (Color online) Effect of varying temperature on J-V curve of simulated bilayer device.

# Device architecture

The solar cell capacitance simulator (SCAPS) is used for device engineering. The structure of the devices in the modeling is FTO / PEDOT:PSS / MAPbI_3_ / FA_0.5_MA_0.5_Pb_0.5_Sn_0.5_I_3_ / PCBM / back metal contact as shown in fig. S5. Also, we should mention that there are IDLs (Interface Defect Layers) among the layers.

_
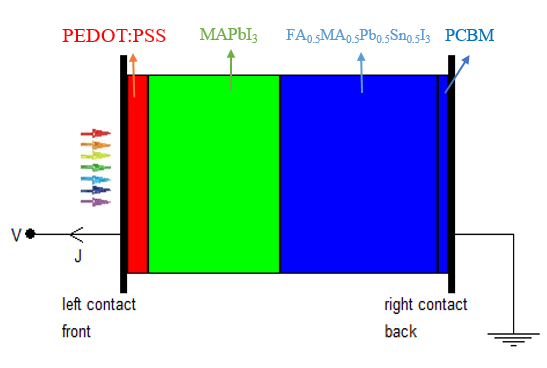
_

**Fig. S5:** (Color online) Schematic of initial bilayer cell in SCAPS.

# Graphical abstract

Fig. S6 shows the graphical abstract of our work. In this figure, the J-V curve, Band diagram, and structure of bilayer solar cell are illustrated.

**
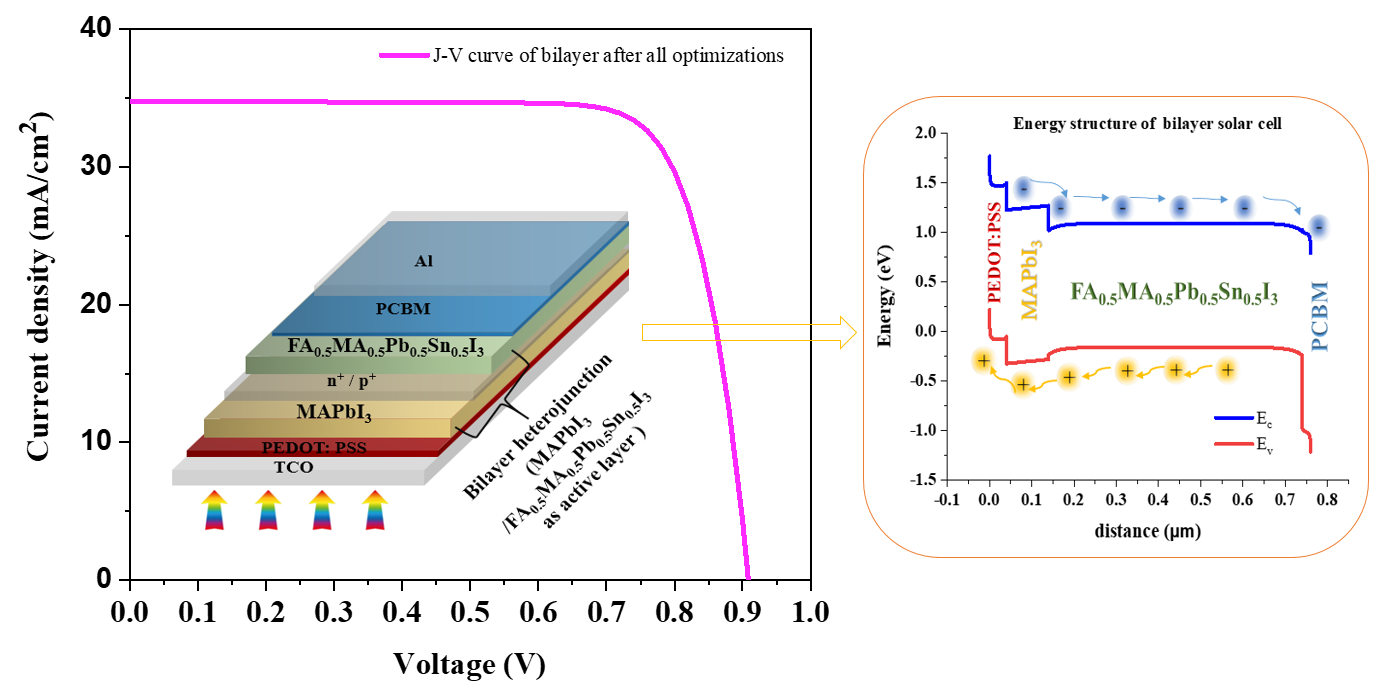
**

**Fig. S6:** (Color online) Graphical abstract: The J-V curve, structure, and Energy structure of inverted all-perovskite bilayer solar cell after all optimizations (at 275 K, the thickness of MAPbI_3_ and FA_0.5_MA_0.5_Pb_0.5_Sn_0.5_I_3_ is 100 nm and 600 nm respectively, and the work-function of front and back contact is 5.4 eV and 4.2 eV, respectively.).

# Validation of the model using wxAMPS

We validated our SCAPS simulation results using a 1-D solar simulator software wxAMPS. It is similar in performance to SCAPS-1D but provides a better ability to model materials with high defect densities, band tails, and other features typical of thin-film solar cells and is written in C++ Language with a number of modifications in basic algorithms, namely, it uses the fundamental physical principles of AMPS-1D, includes portion tunneling currents to the model, delivers an improved visualization and, improves convergence and execution speed [4,5]. wxAMPS is a high-performance software to simulate the behaviors of heterojunction solar cells [6].

Fig. S7(a) and Fig. S7(b) indicate the comparison in J-V and QE curves between the two simulation tools. Table S2 shows that the values of wxAMPS are very similar to the values obtained from SCAPS-1D simulation for the proposed solar cell.

**Table S2:** Comparison between the SCAPS and wxAMPS results for the optimized bilayer solar cell.

| Software/parameters | PCE (%) | FF (%) | J_sc_ (mA/cm^2^) | V_oc_ (V) |
| --- | --- | --- | --- | --- |
| SCAPS-1D | 24.83 | 79.01 | 34.7649 | 0.9091 |
| wxAMPS | 25.04 | 84.68 | 32.7122 | 0.9112 |

**Fig. S7:** (Color online) Comparison in the (a) J-V and (b) QE curves of the optimized bilayer solar cell between the wxAMPS and SCAPS software.

**REFERENCES**

[1] A. Slami, N. Benramdane, Manual method for measuring the external quantum efficiency for solar cells, in: E3S Web Conf., 2021: pp. 1–5. https://doi.org/10.1051/e3sconf/202122901005.

[2] H. Li, C. Zhang, D. Li, Y. Duan, Simulation of transform for external quantum efficiency and power efficiency of electroluminescent devices, J. Lumin. 122–123 (2007) 626–628. https://doi.org/10.1016/j.jlumin.2006.01.243.

[3] P. Singh, N.M. Ravindra, Temperature dependence of solar cell performance - An analysis, Sol. Energy Mater. Sol. Cells. 101 (2012) 36–45. https://doi.org/10.1016/j.solmat.2012.02.019.

[4] M. Hadjab, J.M. Wagner, F. Bouzid, S. Boudour, A. Hadj Larbi, H. Bennacer, M.I. Ziane, M.A. Saeed, H. Abid, S. Berrah, A numerical optimization study of CdS and Mg0.125Zn0.875O buffer layers in CIGS-based solar cells using wxAMPS-1D package, Int. J. Model. Simul. 42 (2022) 179–191. https://doi.org/10.1080/02286203.2020.1857129.

[5] Y. Liu, Y. Sun, A. Rockett, A new simulation software of solar cells - WxAMPS, Sol. Energy Mater. Sol. Cells. 98 (2012) 124–128. https://doi.org/10.1016/j.solmat.2011.10.010.

[6] K. Shivesh, I. Alam, A.K. Kushwaha, M. Kumar, S.V. Singh, Investigating the theoretical performance of Cs2TiBr6-based perovskite solar cell with La-doped BaSnO3 and CuSbS2 as the charge transport layers, Int. J. Energy Res. 46 (2022) 6045–6064. https://doi.org/10.1002/er.7546.
